# Comparing Vadadustat and Darbepoetin in Maintenance Dialysis with CKD-Related Anemia: A Win Statistics Analysis

**DOI:** 10.1681/ASN.0000001090

**Published:** 2026-03-17

**Authors:** Glenn M. Chertow, Rajiv Agarwal, Steven K. Burke, Wenli Luo, Todd E. Minga, Mark J. Sarnak, Wolfgang C. Winkelmayer, Kai-Uwe Eckardt

**Affiliations:** 1 Stanford University School of Medicine, Stanford, California; 2 Indiana University School of Medicine, Indianapolis, Indiana; 3 Akebia Therapeutics, Inc., Cambridge, Massachusetts; 4 Tufts University School of Medicine, Boston, Massachusetts; 5 Baylor College of Medicine, Houston, Texas; 6 Charite – Universitatsmedizin Berlin, Berlin, Germany

**Keywords:** anemia, CKD, clinical trial, dialysis, hospitalization, mortality

## Introduction

Vadadustat is an oral hypoxia-inducible factor prolyl hydroxylase inhibitor that stimulates endogenous erythropoietin and red blood cell production.^1^ In the United States, vadadustat is approved for the treatment of anemia due to CKD in adults receiving dialysis for at least 3 months.^1^ Safety and efficacy were compared with darbepoetin alfa, an erythropoiesis-stimulating agent, in two phase 3, global, open-label, active-controlled noninferiority trials in adults with anemia associated with dialysis-dependent CKD (INNO_2_VATE).^2^

In the primary analysis, vadadustat was determined to be noninferior to darbepoetin alfa for time to first major adverse cardiovascular event, a composite end point of all-cause mortality, nonfatal myocardial infarction, or nonfatal stroke (hazard ratio, 0.96; 95% confidence interval [CI], 0.83 to 1.11)^2^ using proportional hazards (“Cox”) regression. Here, we conducted a *post hoc* win statistics analysis^3^ of the INNO_2_VATE trials to account for all events and to prioritize end points—namely, mortality and hospitalization, an exploratory end point—to better assess the integrated effects of vadadustat on these meaningful outcomes.

## Methods

The design and primary results of the INNO_2_VATE trials have been previously reported.^2^ Eligible patients were randomized 1:1 to vadadustat at a starting dose of 300 mg orally daily, or darbepoetin alfa administered according to the previous dose or local product label. Doses of vadadustat and darbepoetin alfa were titrated to maintain region-specific target hemoglobin concentrations (in the United States, 10–11 g/dl; other countries, 10–12 g/dl). The primary safety outcome of the primary analysis was time to first major adverse cardiovascular event.

This *post hoc* analysis was conducted among all randomized patients who received at least one dose of study drug (safety population). A hierarchical composite end point of (*1*) time to all-cause mortality and (*2*) hospitalization with consideration of exposure time was analyzed using win statistics. Each patient in the vadadustat group was compared with each patient in the darbepoetin alfa group first on time to death, and if they survived through study follow-up, on hospitalization second. Each paired comparison resulted in a win, loss, or tie. We calculated the win ratio by dividing all wins from the vadadustat group by wins from the darbepoetin alfa group. To account for ties, we calculated the win odds by dividing the number of wins plus half of the ties in the vadadustat group by the number of wins plus half of the ties in the darbepoetin alfa group. The 95% CI and *P* values were estimated using the Finkelstein–Schoenfeld test. We performed sensitivity analyses of all patients on treatment plus 28 days to include mortality and hospitalizations during active drug exposure. Hospitalizations were captured during the trial as serious treatment-emergent adverse events and coded by system organ class according to MedDRA v23.0. Additional details and expert perspectives on win analyses have been published previously.^3–6^

## Results and Discussion

Overall, 3902 patients were included in the safety population in the INNO_2_VATE trials: 1947 received vadadustat (3222.0 patient-years [PY]) and 1955 received darbepoetin alfa (3245.8 PY). The incidence and rate of all-cause mortality was 14.9% and 9.0 events per 100 PY for vadadustat (291/1947 patients; 291 events) and 15.9% and 9.6 events per 100 PY for darbepoetin alfa (310/1955; 310 events). The incidence and rate of hospitalizations was 50.6% and 101.8 events per 100 PY for vadadustat (986/1947 patients; 3279 events) and 53.9% and 110.3 events per 100 PY for darbepoetin alfa (1053/1955 patients; 3580 events). The most common serious treatment-emergent adverse events that required or prolonged hospitalization were in the infections and infestations category with incidences, events, and event rates of 26.2% (511/1947 patients), 865 events, and 26.8 events per 100 PY, respectively, for vadadustat and 26.9% (526/1955 patients), 942 events, and 29.0 events per 100 PY, respectively, for darbepoetin alfa. The second most common category was cardiac disorders with incidences, events, and event rates of 13.0% (253/1947 patients), 420 events, and 13.0 events per 100 PY, respectively, for vadadustat and 16.2% (317/1955 patients), 510 events, and 15.7 events per 100 PY, respectively, for darbepoetin alfa.

**Figure 1:**
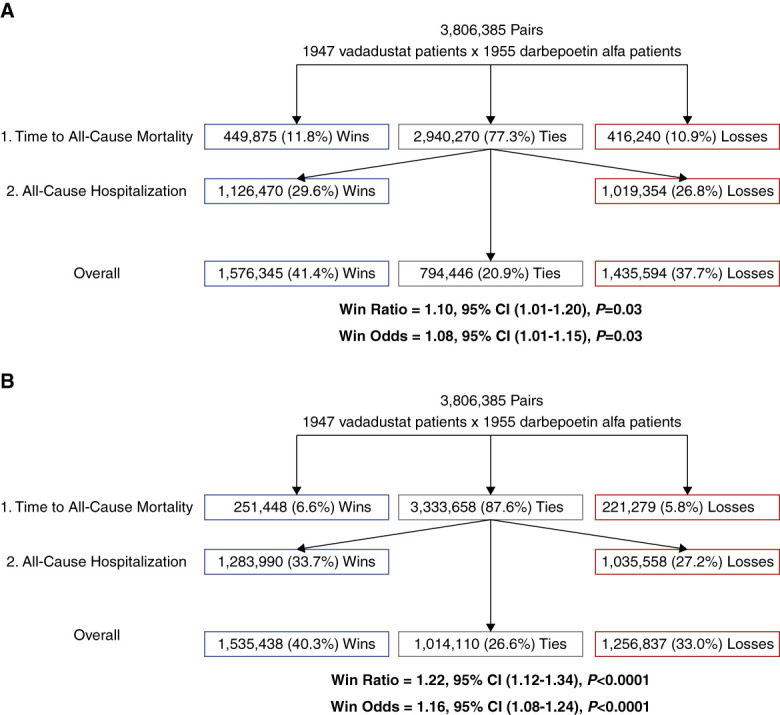
**Lower likelihood of death or hospitalization with vadadustat.** Win ratio and win odds analyses for the composite of all-cause mortality and hospitalizations (A) on-study and (B) on-treatment plus 28 days. Win: a better outcome for vadadustat than darbepoetin alfa; loss: a worse outcome for vadadustat than darbepoetin alfa; tie: the same outcome for vadadustat and darbepoetin alfa. The outcomes were based on the predefined hierarchy of all-cause mortality, and if both patients survived through study follow-up (tie), then on all-cause hospitalization. CI, confidence interval.

The win statistics analyses included a total of 3,806,385 patient pairs. For the composite of all-cause mortality and hospitalizations, the win count in the vadadustat group was 1,576,345, and the win count in the darbepoetin alfa group was 1,435,594. The win ratio (95% CI) was 1.10 (1.01 to 1.20) and the win odds (95% CI) 1.08 (1.01 to 1.15), both nominally significant and in favor of vadadustat (Figure 1A). In the on-treatment analysis, the win ratio (95% CI) was 1.22 (1.12 to 1.34) and the win odds (95% CI) 1.16 (1.08 to 1.24), both nominally significant and in favor of vadadustat (Figure 1B).

We had previously reported a win statistics analysis of the INNO_2_VATE primary composite end point, prespecifying the end point hierarchy as death, followed by nonfatal stroke, followed by nonfatal myocardial infarction.^7^ The win ratio (95% CI) was 1.07 (0.92 to 1.25) and win odds 1.02 (0.97 to 1.08), which indicated favorable trends but did not reach statistical significance.

In this *post hoc* analysis of the INNO_2_VATE trials, we employed hierarchical win ratio and win odds analyses for (*1*) time to all-cause mortality and (*2*) hospitalization as a composite end point. Among patients with dialysis-dependent CKD and CKD-related anemia, those randomized to vadadustat experienced lower rates of the composite end point of all-cause mortality or hospitalization compared with patients randomized to darbepoetin alfa.

## Supplementary Material

## Data Availability

Original data generated for the study will be made available upon reasonable request to the corresponding author. Data Type: Clinical Trial Data.

## Declarative Statements

This study includes clinical experimentation and received Institutional Review Board or Ethics Committee approval. All patients provided written informed consent. This study includes clinical experimentation and complies with the Declaration of Helsinki.

## References

[1] Vafseo^®^ [Package Insert]. Akebia Therapeutics, Inc., 2024.

[2] Eckardt KU Agarwal R Aswad A, . Safety and efficacy of vadadustat for anemia in patients undergoing dialysis. N Engl J Med. 2021;384(17):1601–1612. doi:10.1056/NEJMoa2025956 33913638

[3] Gregson J Taylor D Owen R Collier T Cohen DJ Pocock S. Hierarchical composite outcomes and win ratio methods in cardiovascular trials: a review and consequent guidance. Circulation. 2025;151(22):1606–1619. doi:10.1161/circulationaha.124.070251 40455842

[4] Wittes J. Reflections on the win ratio with time-to-event outcomes. Circ Heart Fail. 2024;17(9):e012186. doi:10.1161/CIRCHEARTFAILURE.124.012186 39193766

[5] Butler J Stockbridge N Packer M. Win ratio: a seductive but potentially misleading method for evaluating evidence from clinical trials. Circulation. 2024;149(20):1546–1548. doi:10.1161/CIRCULATIONAHA.123.067786 38739696

[6] Pocock SJ Gregson J Collier TJ Ferreira JP Stone GW. The win ratio in cardiology trials: lessons learnt, new developments, and wise future use. Eur Heart J. 2024;45(44):4684–4699. doi:10.1093/eurheartj/ehae647 39405050 PMC11578645

[7] Chertow GM Burke S Luo W, . A Win-Ratio Analysis of the Cardiovascular Safety of Vadadustat in Patients with CKD-Related Anemia Undergoing Dialysis. 62nd European Renal Association (ERA) Congress 2025.

